# Oestrogen promotes tumorigenesis of bladder cancer by inducing the enhancer RNA—eGREB1

**DOI:** 10.1111/jcmm.13861

**Published:** 2018-09-04

**Authors:** Mengting Ding, Yuhan Liu, Jianfa Li, Lin Yao, Xinhui Liao, Haibiao Xie, Kang Yang, Qun Zhou, Yuchen Liu, Weiren Huang, Zhiming Cai

**Affiliations:** ^1^ Department of Urology Shenzhen Second People′s Hospital The First Affiliated Hospital of Shenzhen University Clinical Medicine College of Anhui Medical University Shenzhen China; ^2^ Anhui Medical University Hefei China; ^3^ Department of Urology Shenzhen Second People′s Hospital The First Affiliated Hospital of Shenzhen University Shenzhen China; ^4^ Guangdong and Shenzhen Key Laboratory of Male Reproductive Medicine and Genetics Institute of Urology Peking University Shenzhen Hospital Shenzhen China; ^5^ Department of Urology Peking University First Hospital Institute of Urology Peking University National Urological Cancer Center Beijing China; ^6^ University of South China Hengyang China

**Keywords:** bladder cancer, eGREB1, enhancer RNAs, oestrogen

## Abstract

In recent years, studies have shown that enhancer RNAs (eRNAs) can be transcribed from enhancers. Increasing evidence has revealed that eRNAs play critical roles in the development of various cancers. Oestrogen‐associated eRNAs are closely related to breast cancer. In view of the gender differences in bladder cancer (BCa), we suppose that oestrogen‐associated eRNAs are also involved in tumorigenesis of BCa. In our study, we first demonstrated that eGREB1 derived from the enhancer of an oestrogen‐responsive gene—GREB1 was up‐regulated in BCa tissues, and the expression level of eGREB1 is positively associated with the histological grade and TNM stage of BCa. Knockdown of eGREB1 by CRISPR‐Cas13a could inhibit cell proliferation, migration and invasion and induce apoptosis in BCa cells T24 and 5637. Besides, we exhibited the promoting effect of oestrogen on BCa cells. What's more, down‐regulation of eGREB1 could improve the malignant biological characteristics of BCa cells induced by oestrogen. In conclusion, our data indicated that eGREB1 plays oncogenic role and oestrogen may promote the occurrence and progression of BCa by inducing eGREB1 production. Our findings provide new insights into the prevention of BCa and develop a novel therapeutic target for the treatment of BCa.

## 1 INTRODUCTION

Bladder cancer is the most common malignant neoplasm of the urinary system, with the occurrence of approximately 429 800 new cases and 165 100 deaths worldwide in 2012.^1^ The aetiology of BCa is intricate. Smoking, excessive chemical exposure and chronic inflammation caused by urinary schistosomiasis could all lead to the occurrence of BCa.^1^, ^2^, ^3^, ^4^, ^5^, ^6^Global statistics has shown that sex hormones also play an important role in the initiation and invasion of BCa, as the incidence of BCa in males has increased 3.3 times higher than that of females, but the latter tend to be more aggressive and cause twice mortality than males with BCa.^7^, ^8^ However, the molecular mechanisms of oestrogen in BCa remain elusive.

Enhancers are cis‐regulatory elements with the ability to increase target gene transcription independent of the distance and direction relative to the promoters.^9^ It is generally accepted that enhancers and promoters are physically close together to form enhancer‐promoter looping (E:P looping) to enhance the expression of target genes.^10^, ^11^ In recent years, an amazing discovery revealed more complicated roles of enhancers in gene transcriptional activation. Enhancers could transcribe into enhancer RNAs (eRNAs) uni‐ or bi‐directionally similar to the promoters when respond to various stimulations.^12^, ^13^, ^14^, ^15^, ^16^ Kim et al^17^ first discovered extensive transcription of neuronal activity‐regulated enhancers. Li et al^16^ demonstrated that oestrogen can induce eRNA production and eRNAs can regulate gene transcription by stabilizing E:P looping in breast cancer cell line MCF‐7. It suggests that eRNAs are functionally crucial in gene transcription.

GREB1, gene regulated in breast cancer 1, is a chromatin‐binding coactivator that stabilizes the combination of oestrogen receptor (ER) and other cofactors.^18^ Gosh et al^19^ were the first to show that GREB1 was an oestrogen regulated gene and played an important role in hormone‐responsive cancer. GREB1 promoted oestrogen‐stimulated proliferation of breast cancer,^19^ prostate cancer^20^and endometriosis.^21^ Oestrogen could induce the transcription of the corresponding enhancers of GREB1, giving rise to GREB1 eRNA (eGREB1) production in MCF‐7.^12^, ^16^ In view of the continuous existence of gender differences in BCa, oestrogen seems to be especially important in BCa progression. It is necessary to elucidate whether eGREB1 participated in the action of oestrogen on BCa.

In this study, we examined eGREB1 expression in BCa tissues and investigated the effect of eGREB1 knockdown on the proliferation, migration, invasion and apoptosis of BCa cells T24 and 5637. Moreover, we explored whether eGREB1 knockdown could influence oestrogen‐induced biological behaviours of BCa cells. Our work aims to clarify the potential molecular mechanisms of oestrogen‐induced BCa and provides new insights for the prevention of BCa.

## 2 MATERIALS AND METHODS

### 2.1 Patient samples

We collected 38 tumour tissues and matched normal bladder tissues from patients diagnosed with bladder urothelial carcinoma. All samples were snap‐frozen in liquid nitrogen immediately after partial or radical cystectomy. We got the written informed consents from all the BCa patients included in this study. The Institutional Review Board of Shenzhen Second People's Hospital approved the study.

### 2.2 Cell culture and treatments

Human bladder cancer cell lines (T24, 5637) were purchased from the America Type Culture Collection (ATCC, Manassas, VA, USA). The T24 cells and 5637 cells were cultured in phenol‐free DMEM medium or phenol‐free RPMI‐1640 medium (Gibco, Thermo Fisher Scientific, Waltham, MA, USA) supplemented with 10% charcoal‐stripped foetal bovine serum (Gibco) and 1% antibiotics (100U/mL penicillin and 100 μg/mL streptomycin sulphates). All cells were placed in a humidified atmosphere of 5% CO_2_ at 37°C. The cells were treated with 10 nM E2 (Sigma) or DMSO (Sigma) for 1 hour to induce eGREB1 expression.

### 2.3 siRNA and CRISPR‐Cas13a vectors transfections

eGREB1 siRNA (si‐eGREB1) and negative control siRNA (si‐NC) were synthesized by Sangon Biotech, Shanghai, China. The sequences were shown: Sense: 5′‐CAGAGAGAUUCAAGCUUGACGGAAU‐3′; Antisense: 5′‐AUUCCGUCAAGCUUGAAUCUCUCUG‐3′.^16^ T24 and 5637 were cultured in 6‐well plates. When grown to 40%‐50% confluence, they were transiently transfected with 50 nM si‐eGREB1 or si‐NC using Lipofectamine 3000 Transfection Reagent (Invitrogen, Carlsbad, CA, USA) according to the protocol.

CRISPR‐Cas13a vectors targeting eGREB1 (Cas13a‐eGREB1) and negative control (Cas13a‐NC) were designed and constructed by Syngen Tech Co., Beijing, China. T24 and 5637 were cultured in 6‐well plates and were transfected with 2.5ug Cas13a‐eGREB1 or Cas13a‐NC when they were grown to 80%‐90% confluence as before.

### 2.4 Real‐time quantitative PCR (RT‐qPCR)

Total RNA was extracted using TRIzol reagent (Invitrogen, Carlsbad, CA, USA) according to the manufacturer's instructions. cDNA was converted from total RNA using a Revertra Ace qPCR RT Kit (Toyobo, Osaka, Japan) according to the instructions. We use GAPDH as the internal control for RT‐qPCR. The primer sequences were as follows: eGREB1 primers forward: 5′‐GCTAACCATGCTGCAAATGA‐3′ and reverse: 5′‐ACACAGTCAGGGCAAAGGAC‐3′^16^; GAPDH primers forward: 5′‐AACGGATTTGGTCGTATTG‐3′ and reverse: 5′‐GGAAGATGGTGATGGGATT‐3′. RT‐qPCR was carried out using real‐time PCR Master Mix (Toyobo) according to the manufacturer's protocols under conditions of 40 cycles of 15 sec at 95°C, 15 sec at 60°C and 45 sec at 75°C through an ABI PRISM 7500 Fluorescent Quantitative PCR System (Applied Biosystems, Foster City, CA, USA). Expression fold changes were calculated using the 2^−▵▵ct^ method.

### 2.5 Cell proliferation assay

Cell Counting Kit‐8, CCK‐8 (TransGen, Beijing, China) was used to detect cell proliferation. After transfection with CRISPR‐Cas13a vectors for 24 h, 5 × 10^3^ cells were seeded in a 96‐well plate. At 0, 24, 48 and 72 h, 10 μL CCK‐8 was added to each plate and incubated at 37°C for 1 h. The absorbance at 450 nm was measured by a microplate reader (Bio‐Rad, Hercules, CA, USA).

### 2.6 Wound healing assay

T24 and 5637 cells were cultured and transfected with either Cas13a‐eGREB1 or Cas13a‐NC for 24 h. A sterile 200 μL pipette tip was used to create clear lines and took pictures immediately. Then, cells were cultured in mediums supplemented with 1% FBS. Twenty‐four hours later, pictures were taken again. Migration area was measured at the time points of 0 and 24 h. Each test was carried out at least three times.

### 2.7 Transwell assay

The cells were treated with Cas13a‐eGREB1 or Cas13a‐NC for 24 h.About 5 × 10^4^ T24 cells and 1.5 × 10^5^ 5637 cells supplemented with 200 μL serum‐free medium were plated into the upper chambers (24‐well insert, pore size 8 μm, Corning) containing matrigel (1:8, 50 μL/well, BD Bioscience, San Jose, CA, USA). The lower chambers were added with 500 μL medium supplemented with 10% FBS. Cells were cultured for 24 hours, and then, cells under the surface of the lower chamber were washed with 1× PBS, fixed with methanol for 20 min, stained with 0.1% crystal violet for 25 min and washed 3 times. Invaded cells were observed under the inverted microscope and imaged. After that, each chamber with the invaded cells was soaked into 1 mL 33% acetic acid for 10 min to wash out the crystal violet. One hundred microlitres of 33% acetic acid was added into each well of 96‐well plates, and then, the absorbance was measured at 570 nm by a microplate reader (Bio‐Rad, Hercules, CA, USA). Experiments were performed in triplicate.

### 2.8 Caspase 3 ELISA assay

Bladder cancer 5637 and T24 cells were transfected with Cas13a vectors in petri plates. Twenty‐hour hours after transfection, apoptosis was detected by measuring the activity of caspase 3 using the caspase 3 enzyme‐linked immunosorbent assay (ELISA) assay kit (Cusabio, Wuhan, China) according to the manufacturer's instructions. OD values were measured at 450 nm using a microplate reader (Bio‐Rad).

### 2.9 Statistical analysis

All data were presented as mean ± standard deviation (SD). The eGREB1 expression differences between BCa tissues and matched normal tissues were analysed using paired samples *t* test. The CCK‐8 assay was analysed using ANOVA. Other data were analysed by the independent samples *t* test. All these statistical analyses were performed using SPSS 17. A *P* value of less than 0.05 was considered to be statistically significant.

## 3 RESULTS

### 3.1 eGREB1 was up‐regulated in BCa tissues and it was related to clinical‐pathological features

**Figure 1:**
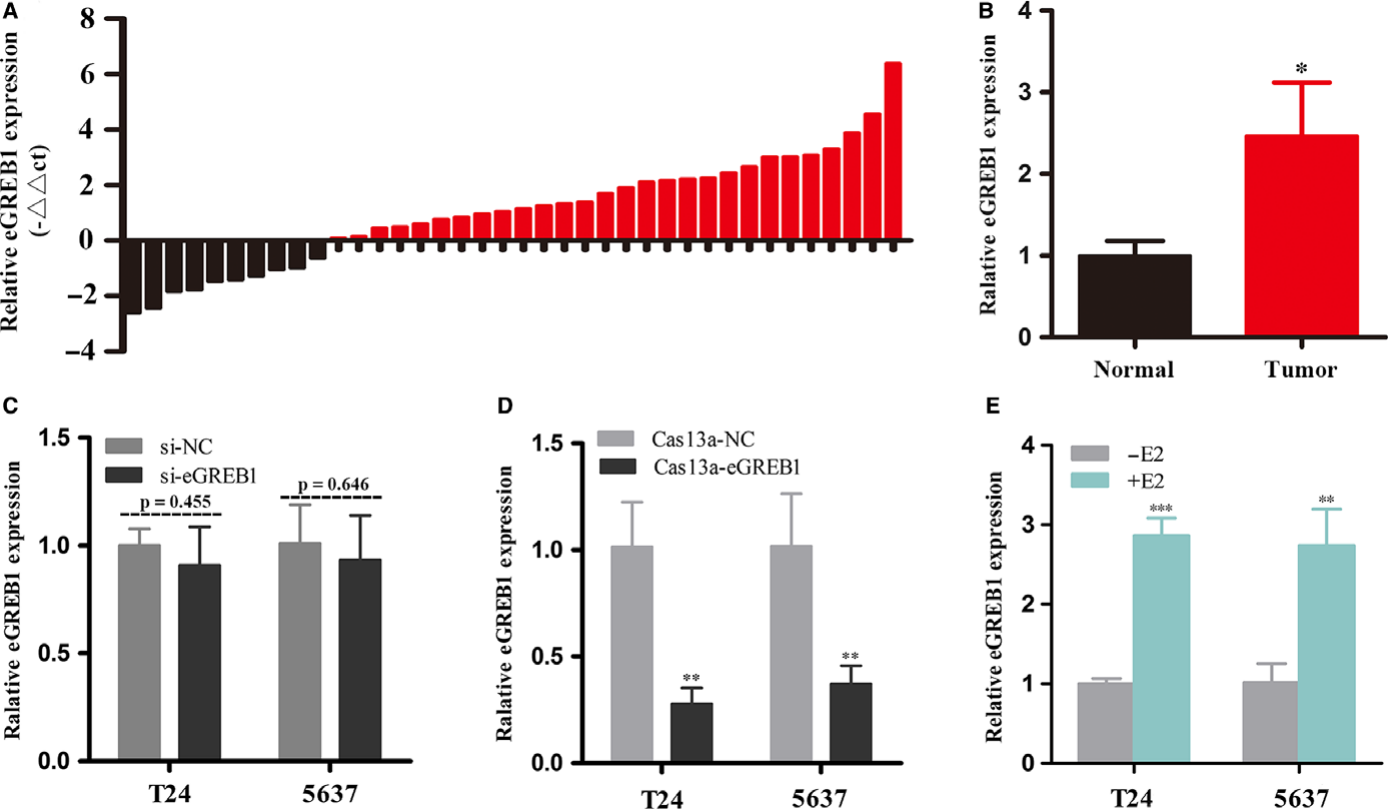
(A) The relative expression levels of eGREB1 were measured by RT‐qPCR in 38 BCa patients. (B) The expression levels of eGREB1 were increased in BCa tissues compared to paired normal tissues. (C) There is no significant change in eGREB1 expression after transfection of siRNA. (D) eGREB1 expression was significantly down‐regulated by Cas13a‐eGREB1. (E) After stimulation with oestrogen for 1 h, the relative expression of eGREB1 was remarkably increased in BCa cells. Data are shown as mean ± SD (*P < 0.05, **P < 0.01, ***P < 0.001)

The relative expression level of eGREB1 in a total of 38 patients with BCa was measured by RT‐qPCR. Compared with matched normal tissues, eGREB1 is significantly up‐regulated in 73.7% (28 of 38) of BCa tissues (*P* = 0.018, Figure 1A, B), indicating the potential role of eGREB1 in facilitating BCa development. Then, we assessed the relationship between eGREB1 expression and patients’ clinicopathological features. As shown in Table 1, there is a positive correlation between the eGREB1 expression level and the clinical‐pathological features including histological grade, depth of invasion and TNM stage of BCa. However, gender, age and lymph node metastasis had no relation with the eGREB1 expression level gender. These results indicated that eGREB1 should function as tumour promoters in BCa.

**Table 1:** Correlation between eGREB1 expression and clinicopathological characteristics of BCa patients

Characteristics	Total	eGREB1 expression		*P* value
		Low	High	
Gender				
Female	7	1 (14.3%)	6 (85.7%)	0.517
Male	31	8 (25.8%)	23 (74.2%)	
Age				
≤60	10	4 (40.0%)	6 (60.0%)	0.252
>60	28	6 (21.4%)	22 (78.6%)	
Histological grade				
Low	15	10 (66.7%)	5 (33.3%)	0.001**
High	23	3 (13.0%)	20 (87.0%)	
Depth of invasion (T)				
Ta, T1	9	7 (77.8%)	2 (22.2%)	0.004**
T2, T3, T4	29	7 (24.1%)	22 (75.9%)	
Lymph node metastasis (N)				
N0	36	7 (19.4%)	29 (80.6%)	0.490
N1, N2, N3	2	0 (0.0%)	2 (100.0%)	
TNM stage				
0/I	6	4 (66.7%)	2 (33.3%)	0.003**
II/III/IV	32	4 (12.5%)	28 (87.5%)	

*P < 0.05 was considered significant; **P < 0.01.

### 3.2 Knockdown of eGREB1 by CRISPR‐Cas13a was far more effective than that induced by siRNA

To compare eGREB1 knockdown efficiency between CRISPR‐Cas13a and siRNA, we measured the relative expression level of eGREB1 at 24 hours after transfection of siRNA or CRISPR‐Cas13a. eGREB1 expression levels did not decrease significantly after transfection of siRNA (Figure 1C, *P* = 0.455 in T24; *P* = 0.646 in 5637). However, for the cells transfected with CRISPR‐Cas13a, the relative expression levels of eGREB1 were reduced to 27.71% and 37.06% in T24 and 5637 cell lines, respectively (Figure 1D, *P* < 0.01 in T24 and 5637). Thus, only CRISPR‐Cas13a is suitable for the knockdown study on eGREB1.

### 3.3 Oestrogen induced the production of eGREB1 in BCa cells

To explore whether oestrogen could induce eGREB1 transcription in BCa cells, we analysed eGREB1 expression after E2 stimulation for 1 h in T24 and 5637. Our results showed the relative expression level of eGREB1 increased by 2.87‐fold in T24 (*P* < 0.001) and 2.74‐fold in 5637 (*P* < 0.01), respectively (Figure 1E). We demonstrated that eGREB1 expression was significantly raised in BCa cells when stimulated by oestrogen.

### 3.4 eGREB1 knockdown inhibited cell proliferation, migration, invasion and promoted apoptosis in BCa cells

**Figure 2:**
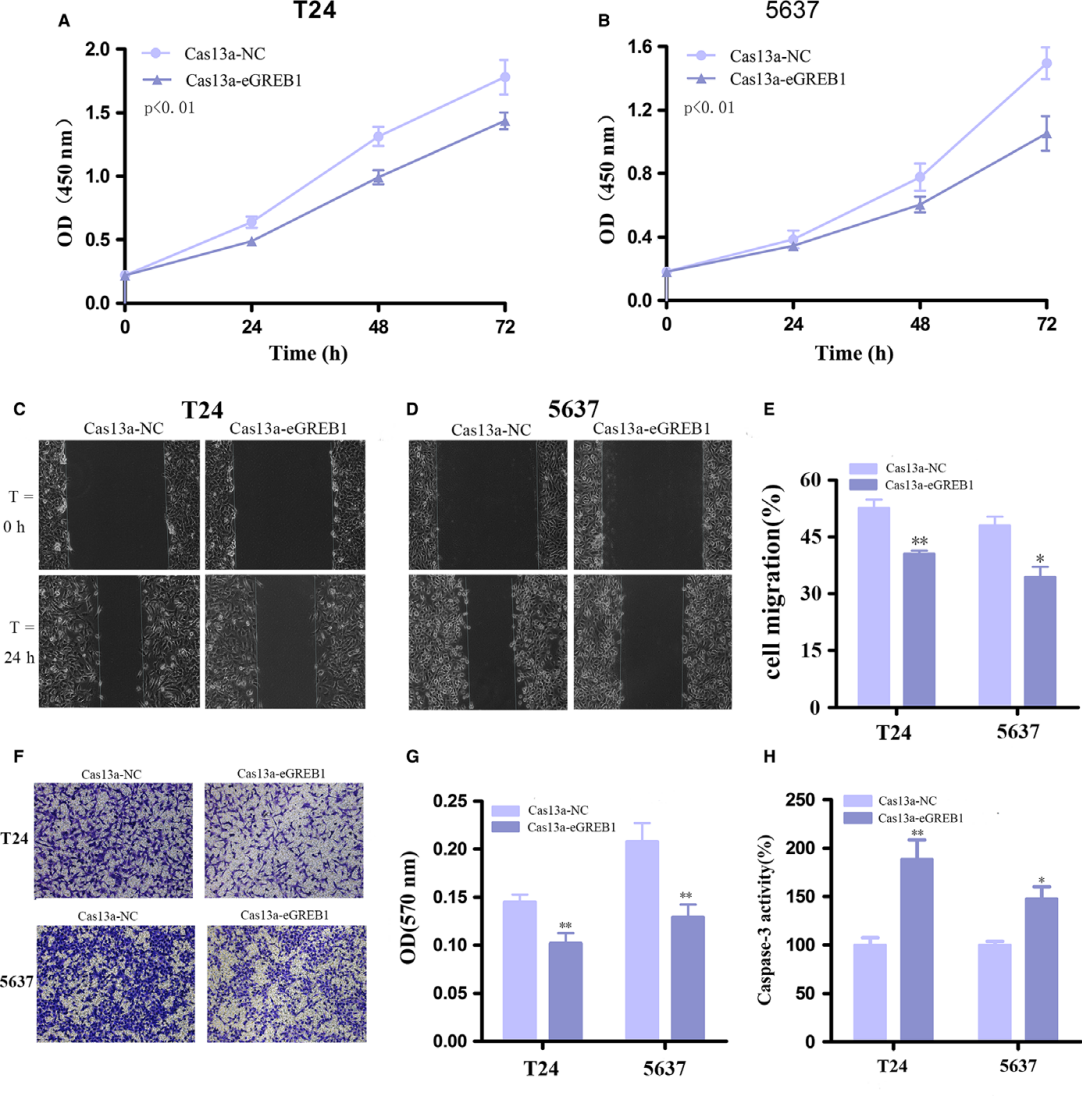
(A, B) Cell proliferation was detected by CCK8 in T24 and 5637 after transfection for 24 h. Knockdown of eGREB1 by Cas13a inhibited cell growth of BCa cells. (C‐E) Wound healing assay was used to measure cell migration. Down‐regulation of eGREB1 suppressed migration of BCa cells. (F, G) Down‐regulation of eGREB1 decreased cell invasion detected by transwell assays. (H) Down‐regulation of eGREB1 increased cell apoptosis measured by Caspase 3 ELISA assay. Experiments were performed in triplicate. Data were shown as mean ± SD (*P < 0.05, **P < 0.01)

Next, we investigated the effect of eGREB1 knockdown on the biological characteristics of BCa cells. After transfection with Cas13a‐eGREB1 for 24 h, we used CCK8 to compare the cell proliferation rates between the eGREB1 knockdown group and the control group. The results suggested eGREB1 knockdown prominently impaired cell growth in T24 (*P* < 0.01) and 5637 (*P* < 0.01) (Figure 2A, B). Wound healing assay was used to compare the ability of migration between the two groups. In contrast, the knockdown group's migration ratio decreased by 12.07% in T24 (*P* = 0.001) and 13.61% in 5637 (*P* < 0.01), respectively (Figure 2C‐E). Besides, transwell assays showed that down‐regulation of eGREB1 could decrease cell invasion of BCa cells (Figure 2F, G, *P* < 0.01 in T24 and 5637). Furthermore, we explored whether knockdown of eGREB1 could facilitate cell apoptosis by Caspase 3 ELISA assay. The results showed that eGREB1 knockdown increased the apoptosis rate of BCa cells (Figure 2H, *P* < 0.01 in T24, *P* < 0.05 in 5637). These results indicated the potential role of eGREB1 in promoting BCa.

### 3.5 eGREB1 knockdown attenuated the cancer‐promoting effect of oestrogen

**Figure 3:**
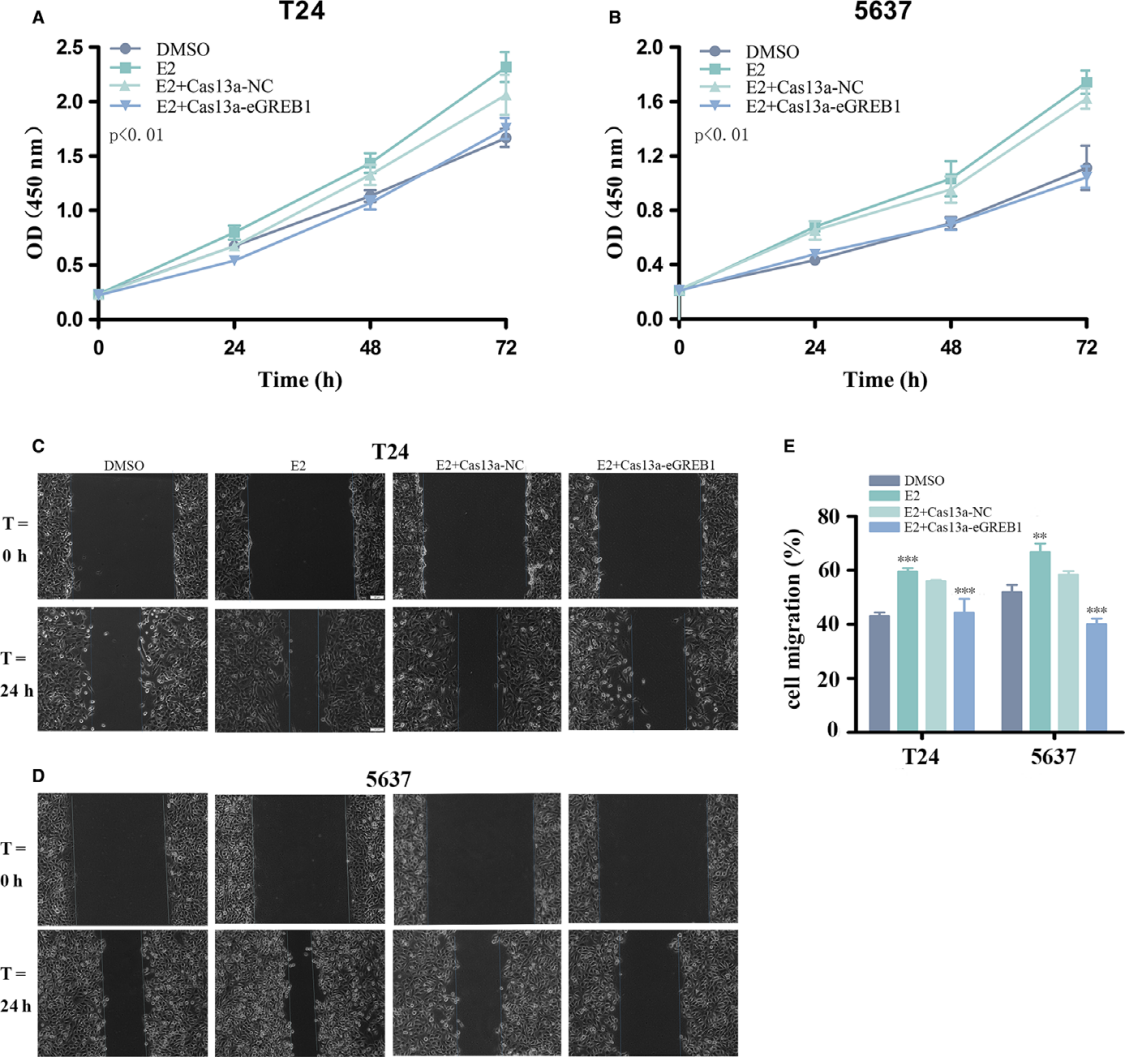
(A, B) Oestrogen promoted cell proliferation and eGREB1 knockdown reduced the pro‐proliferative effects of oestrogen in T24 and 5637. (C‐E) Oestrogen facilitated cell migration of T24 and 5637. eGREB1 knockdown impaired cell migration induced by oestrogen. Experiments were performed in triplicate. Data were shown as mean ± SD (*P < 0.05, **P < 0.01, ***P < 0.001)

**Figure 4:**
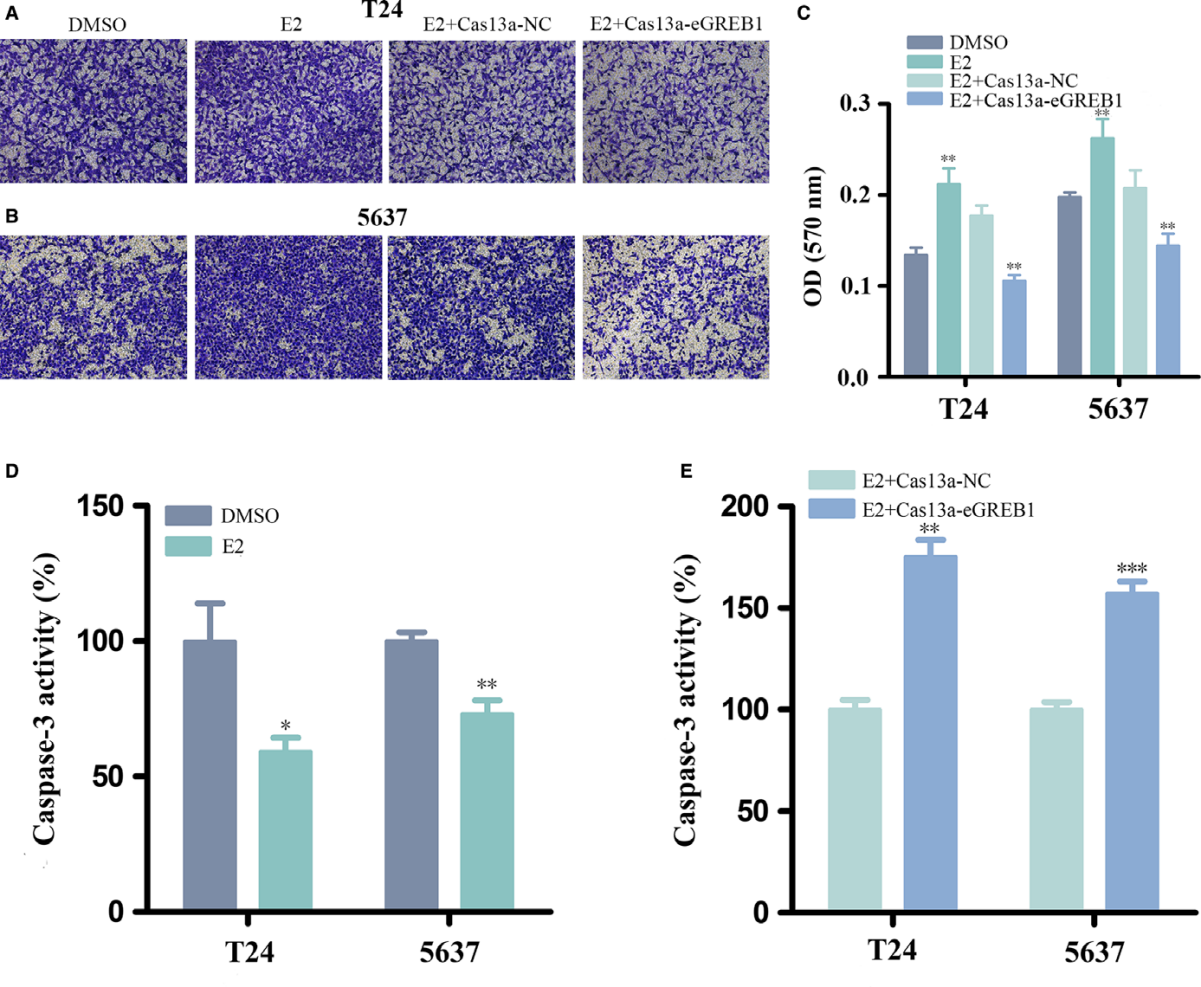
(A‐C) Oestrogen promoted the proliferation and down‐regulation of eGREB1 attenuated oestrogen‐induced invasion of BCa cells. (D) O**e**strogen reduced BCa cell apoptosis. (E) eGREB1 knockdown with oestrogen treatment increased cell invasion compared to the control group. Experiments were performed in triplicate. Data were shown as mean ± SD (*P < 0.05, **P < 0.01, ***P < 0.001)

We revalidated the cancer‐promoting effect of oestrogen on BCa. Oestrogen promoted the cell proliferation (Figure 3A, B, *P* < 0.01 in and 5637), migration (Figure 3C‐E, *P* < 0.001 in T24, *P* < 0.01 in 5637), invasion (Figure 4A‐C, *P* < 0.01 in T24 and 5637) and inhibited apoptosis (Figure 4D, *P* < 0.05 in T24, *P* < 0.01 in 5637).

Subsequently, BCa cells were transfected with Cas13a‐eGREB1 or Cas13a‐NC vectors and treated with oestrogen meanwhile. We compared their biological behaviours using the methods described above. For the group of eGREB1 knockdown with oestrogen treatment, the cell proliferation was obviously decreased (Figure 3A, B, *P* < 0.01 in T24 and 5637); the cell migration ratio were reduced by 11.68% in T24 and 18.28% in 5637 (Figure 3C‐E, *P* < 0.001 in T24 and 5637); the invasive ability of the cells is also prominently weakened (Figure 4A‐C, *P* < 0.01 in T24 and 5637); ELISA assay showed that the activity of caspase‐3 were increased (Figure 4E, *P* = 0.001 in T24, *P* < 0.001 in 5637). In conclusion, eGREB1 knockdown can attenuate the effect of oestrogen on BCa.

## 4 DISCUSSION

Increasingly evidences implicate that enhancers are functionally important in key cellular processes including cell development, differentiation and apoptosis. eRNAs are generated by the transcription of active enhancers and involve in the development of various diseases by regulating the expression of multiple genes.^22^, ^23^, ^24^, ^25^, ^26^ GREB1 is shown to be one of the most oestrogen‐specific genes and plays an important role in oestrogen‐mediated transcriptional activation. The corresponding enhancer of GREB1 could transcribe into eGREB1 when exposed to oestrogen. However, the role of eGREB1 in BCa is unclear. There have been reported that oestrogen is of great importance in the biologic aggressiveness of BCa.^27^, ^28^ Anti‐oestrogen therapies could inhibit BCa progression.^29^, ^30^ Whether oestrogen promotes BCa progression through promoting eGREB1 transcription is yet not known.

This is the first study to demonstrate the overexpression of eGREB1 in BCa tissues. High level expression of eGREB1 was associated with high grade and TNM stage of BCa. Differences in eGREB1 expression of BCa and control and the connection of eGREB1 with clinicopathological features suggest that it becomes a newcomer to the initiate and progression of BCa.

Rahman et al^31^ have shown that eRNAs were merely located in the nucleus in MCF‐7 cells. Current research results show that eRNAs could enhance gene transcription through multiple ways, such as acting on E:P looping^14^, ^32^, ^33^ or recruiting RNA polymerase.^34^, ^35^, ^36^ It seems that eRNAs play their roles only in the nucleus. Although several studies used siRNA to mediate knocked down of eRNAs to explore their biological functions and got significant achievements,^13^, ^14^, ^16^, ^37^ our results show that eGREB1 knockdown by siRNA is barely satisfactory. Instead, targeted knockdown of eGREB1 by CRISPR‐Cas13a is extremely meaningful. The Cas13a construct could localize to the nucleus and cleave transcripts precisely and efficiently based on the nuclear localization sequence.^38^ In summary, we demonstrated that CRISPR‐Cas13a is more applicable to knockdown of eRNAs.

To further verify the function of eGREB1 in BCa, we used CRISPR–Cas13a vectors to reduce eGREB1. Cell proliferation inhibition, decreased motility and increased apoptosis were observed in T24 and 5637 cells. These findings indicated that eGREB1 may play positive roles in the occurrence and progression of BCa. Then, we explored the effects of oestrogen on the biological characteristics of BCa and found that oestrogen could promote cell proliferation, migration and invasion and inhibit cell apoptosis, which was consistent with previous studies.^29^, ^39^ Last but most important is that knockdown of eGREB1 is able to reverse oestrogen‐induced biological effects on BCa cells. In this way, we suppose that the cancer‐promoting effect of oestrogen may be achieved at least in part through the induction of eGREB1.

In conclusion, our study demonstrated the crucial role of eGREB1 in BCa and oestrogen may promote the development of BCa by inducing eGREB1 production for the first time. There are many molecular hypotheses of the association between oestrogen and BCa. Current researches mostly have indicated that oestrogen interacts with oestrogen receptors or a membrane protein named GPR30 to damage genome stability to influence BCa progression. The abnormal cell proliferation of BCa could be prevented when inhibition of oestrogen receptor *β* reduced the expression of MCM5 which has the ability to interfere with the initiation and extension of DNA replication. Furthermore, anti‐oestrogen drugs such as raloxifene promoted the synthesis of apoptotic proteins to induce the cell apoptosis of BCa. Different from the above, our research brings forward a new perspective for the molecular mechanisms of oestrogen in BCa and opens up a fresh field for exploring the oestrogen‐induced BCa. In the light of functional roles of eGREB1 in BCa, it may possess potential applications in the prevention and cure of BCa. More in‐depth researches should be implemented in future works.

## ACKNOWLEDGEMENTS

This work was supported by the National Key Basic Research Program of China (973 Program) (2014CB745201), National Natural Science Foundation of China (81772737), the Shenzhen Municipal Government of China (JCYJ20170413161749433, JSGG20160301161836370), the Sanming Project of Shenzhen Health and Family Planning Commission (SZSM201412018, SZSM201512037). The high level university's medical discipline construction 2016031638.

## CONFLICT OF INTEREST

All authors declare that there is no conflict of interests.
